# Unprecedented Epimerization of an Azithromycin Analogue: Synthesis, Structure and Biological Activity of 2′-Dehydroxy-5″-Epi-Azithromycin

**DOI:** 10.3390/molecules27031034

**Published:** 2022-02-03

**Authors:** Goran Kragol, Victoria A. Steadman, Zorica Marušić Ištuk, Ana Čikoš, Martina Bosnar, Dubravko Jelić, Gabrijela Ergović, Marija Trzun, Berislav Bošnjak, Ana Bokulić, Jasna Padovan, Ines Glojnarić, Vesna Eraković Haber

**Affiliations:** 1 Fidelta Ltd., Prilaz baruna Filipovića 29, 10000 Zagreb, Croatia; zorica.marusicistuk@fidelta.eu (Z.M.I.); ana.cikos@irb.hr (A.Č.); martina.bosnar@fidelta.eu (M.B.); dubravko.jelic@fidelta.eu (D.J.); gergovic@gmail.com (G.E.); marija.trzun@hotmail.com (M.T.); bosnjak.berislav@mh-hannover.de (B.B.); ana.bokulic@fidelta.eu (A.B.); jasna.padovan@fidelta.eu (J.P.); ines.glojnaric@fidelta.eu (I.G.); vesna.erakovichaber@fidelta.eu (V.E.H.); 2 GlaxoSmithKline, New Frontiers Science Park, Third Avenue, Harlow CM19 5AW, UK; victoria.s@sailife.com

**Keywords:** macrolides, Barton–McCombie oxidation, azithromycin, anti-inflammatory activity

## Abstract

Certain macrolide antibiotics, azithromycin included, possess anti-inflammatory properties that are considered fundamental for their efficacy in the treatment of chronic inflammatory diseases, such as diffuse pan-bronchiolitis and cystic fibrosis. In this study, we disclose a novel azithromycin analog obtained via Barton–McCombie oxidation during which an unprecedented epimerization on the cladinose sugar occurs. Its structure was thoroughly investigated using NMR spectroscopy and compared to the natural epimer, revealing how the change in configuration of one single stereocenter (out of 16) profoundly diminished the antimicrobial activity through spatial manipulation of ribosome binding epitopes. At the same time, the anti-inflammatory properties of parent macrolide were retained, as demonstrated by inhibition of LPS- and cigarette-smoke-induced pulmonary inflammation. Not surprisingly, the compound has promising developable properties including good oral bioavailability and a half-life that supports once-daily dosing. This novel anti-inflammatory candidate has significant potential to fill the gap in existing anti-inflammatory agents and broaden treatment possibilities.

## 1. Introduction

Many studies have demonstrated that certain macrolide antibiotics are also effective in the treatment of various chronic inflammatory disorders [1,2,3]. However, the long-term use in such indications is still limited mostly due to the risk of increasing the incidence of macrolide-resistant bacterial strains. This could be overcome by specifically designed macrolide analogs where antimicrobial activity is completely abolished by specific chemical modification while, at the same time, the anti-inflammatory and pharmacokinetic properties of the parent macrolide are retained [4,5,6,7]. The fact that the antibacterial and anti-inflammatory activities of macrolides are independent and can be separated by subtle modification of ribosome binding epitopes [4] provides the basis for the development of novel anti-inflammatory agents. Although several other mechanisms of macrolide action are mentioned in the literature [8,9,10,11], the primary mechanism for their antibacterial activity directly reflects the location of macrolide binding to the ribosome. During protein synthesis, a newly created protein exits the ribosome through a tunnel that connects the site of polypeptide assembly (peptidyl transferase center—PTC) and protein release outlet on the “back” side of the 50S subunit [12,13]. Macrolides bind in the vicinity of the PTC and serve as a barricade that impedes the progression of the nascent peptide, thus inhibiting translation. More accurately, they interact primarily with 23S rRNA and lie on the floor of the exit tunnel, occupying a space large enough to block the exit of the nascent peptide.

Building upon our knowledge of macrolide ribosome interactions in solution [14,15], as well as existing X-ray crystal structures of various ribosomes in a complex with known macrolides [16,17,18,19], we summarized the binding epitopes of macrolides to be: desosamine, cladinose and the macrolactone methyl group 15.

The significance of desosamine originates from the analysis of crystal structures and identification of only one hydrogen bond between the macrolide and ribosome, connecting the 2′-hydroxyl group of desosamine and N1 of the A2058Ec of the ribosome [16,17,18,19]. The plausibility of separating the anti-microbial and anti-inflammatory properties via modification of desosamine was demonstrated through the series of N′-substituted 2′-O,3′-N-carbonimidoyl bridged erythromycin-derived 14- and 15-membered macrolides [4].

The importance of cladinose, on the other hand, was demonstrated in solution with NMR spectroscopy, through a drastic decrease in STD NMR enhancements and intensity of trNOESY signals observed for compounds without this moiety [14].

In order to transform the well-known antibiotic azithromycin into a full anti-inflammatory drug without any trace of antimicrobial activity, we intended to completely abolish its ability to bind to the bacterial ribosome. Since modifications of the macrolactone methyl group 15 could not be easily achieved through conventional chemistry approaches, we contemplated modification of the azithromycin at the two other key areas important for ribosome binding: the 2′-hydroxyl group of desosamine and cladinose.

Therefore, here, we present a novel scaffold, obtainable in a one-pot two-step procedure starting from commercially available azithromycin via a Barton–McCombie radical deoxygenation reaction [20,21,22], in which both desosamine and cladinose sugars were simultaneously modified. Besides the anticipated 2′ deoxygenation, a change of stereochemistry at the 5″-position on cladinose sugar also occurred. This novel product proved to have superior properties to the solely 2′-deoxy azithromycin analog. The lack of antibacterial activity and retention of in vivo anti-inflammatory and pharmacokinetic properties of the novel azithromycin derivative are discussed below.

## 2. Results

### 2.1. Chemistry

**Scheme 1:**
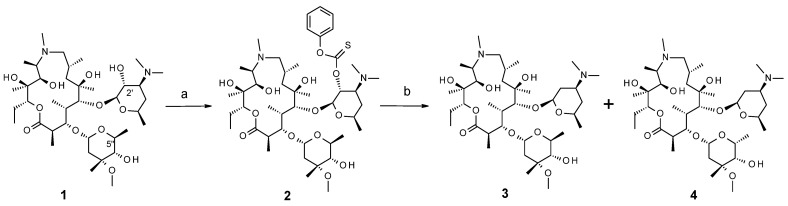
Reagents and conditions: (**a**) *O*-phenyl thionochloroformate, NaHCO_3_, toluene, 60 °C, 2 h; (**b**) see Table 1.

Since the hydroxyl group at position C-2′ of the desosamine sugar is the most reactive site of 14- and 15-membered macrolides, the reaction of azithromycin (**1**) with *O*-phenyl chlorothionoformate produced 2′-phenylthiocarbonate derivative **2** (Scheme 1). We found that sodium hydrogen carbonate/toluene gave a cleaner reaction profile in comparison to triethylamine/toluene. Furthermore, a reaction temperature of 60 °C was found to be optimal. A subsequent radical deoxygenation reaction using typical Barton–McCombie conditions, involving tributyltin hydride/AIBN/toluene (Table 1, entry 1), surprisingly produced a mixture of two 2′-dehydroxy compounds with the same molecular mass in a ratio of 2.5:1. However, if a larger amount of tributyltin hydride was used, only one compound was obtained (Table 1, entry 2). Chromatographic separation of the two products allowed us to clearly identify their chemical structures. It was found that, apart from the expected main product **3**, where radical deoxygenation of the 2′-hydroxy group had occurred, a minor product **4** was also produced, where the inversion of stereochemistry at C-5″ of the cladinose sugar had occurred that was unambiguously confirmed by 2D NMR spectroscopy.

The initial NMR analysis was performed in chloroform-d_1_ yielding full assignment and revealing identical connectivity of **3** and **4**, corroborating that they are stereoisomers. The biggest difference between the two was observed in both proton and carbon chemical shifts of the cladinose moiety, most strikingly of atoms 1″ and 5″. Coupling constants analysis suggested the inversion of stereochemistry at position 5″. Primarily in the interest of resolving the stereochemistry, but also in determining the overall conformation of the newly formed “non-natural” isomer, further investigations were transferred from the chloroform-d_1_ solution to a TRIS-buffered D_2_O media with a pH of 7.4, more similar to living cells. As already observed for the related macrolides [14,23,24], in D_2_O, the NOESY signals intensities are close to zero or change sign, so ROESY spectra were used instead. Not surprisingly, analysis of coupling constants (Table 2) and nOe contacts (Supplementary Materials, Table S1) showed that in both compounds the “western” part of the lactone ring retained the same conformation as in azithromycin [20]. Additionally, despite the absence of the 2′-OH group, the desosamine sugar in both compounds remained in the same chair [25,26] conformation as in azithromycin.

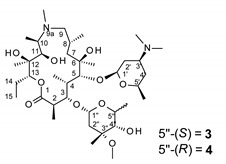



**Figure 1:**
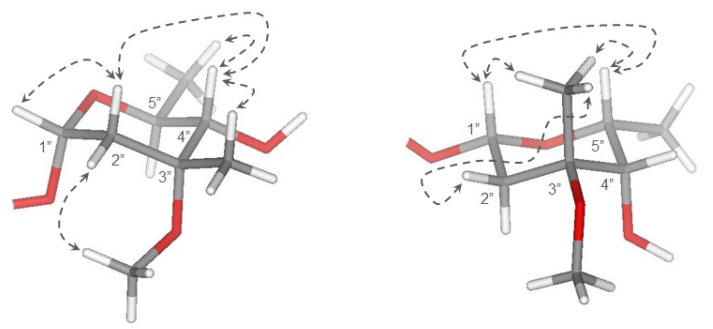
Conformation of cladinose sugar in **3** and **4** with most important nOe interactions (not energy minimized, for illustrative purposes only).

Although cladinose assumes the usual conformation in **3**, in epimer **4** it flips into the inverted chair (Figure 1). Typical strong nOe contact between axial H-4″ and H-2″b is still observed in **3** but is replaced by a system of interactions between axial H-1″/H-3″Me/H-5″ in **4**. These interactions confirm the inversion of configuration at position 5″, reaffirmed also by small (less than 1 Hz) coupling constants between H-4” and H-5″. As a comparison, in **3** this constant is 9.8 Hz. Consequentially, and witnessed by the axial position of H-1” (^3^*J*_H-1”,H-2”b_ = 11.0), cladinose in **4** does not seem to be stabilized by the anomeric effect.

This “new” conformation of cladinose in **4** affects the mutual arrangement of the sugars, as well as their orientation with respect to the lactone ring. Unlike in both **3** and azithromycin, the sugars become more spatially removed which is noticeable by the disappearance of nOe cross-peaks between the two moieties. Consequentially, the lactone ring exhibits outward folding of the C-3 to C-5 region into “folded-out” conformation, characterized by a large ^3^*J*_H-2,H-3_ = 11 Hz and very strong nOe interaction between protons H-4 and H-11.

Having shown that we could overturn the initial ratio of 2.5:1 **3**:**4** to afford only **3** using a larger number of equivalents of tributyltin hydride, we then wished to address the question of overturning the ratio to afford predominantly **4**. Exchanging tributyltin hydride with tris(trimethylsilyl)silane favored the formation of epimer **4** (entry 3). The formation of **4** is favored still further by decreasing the concentration of the reaction mixture (entry 4). Moreover, exchange of the AIBN (1,1′-azobis(*i*-butylcarbonitrile)) with less toxic ACCN (1,1′-azobis(cyclohexanecarbonitrile)) further increased the ratio of **4** (entry 5). After further optimization of the tris(trimethylsilyl)silane/ACCN reaction conditions, it was found that the best ratio of **4** was obtained if 0.5 eq of ACCN was used and when the reaction temperature was increased to 115 °C (entry 6).

**Scheme 2:**
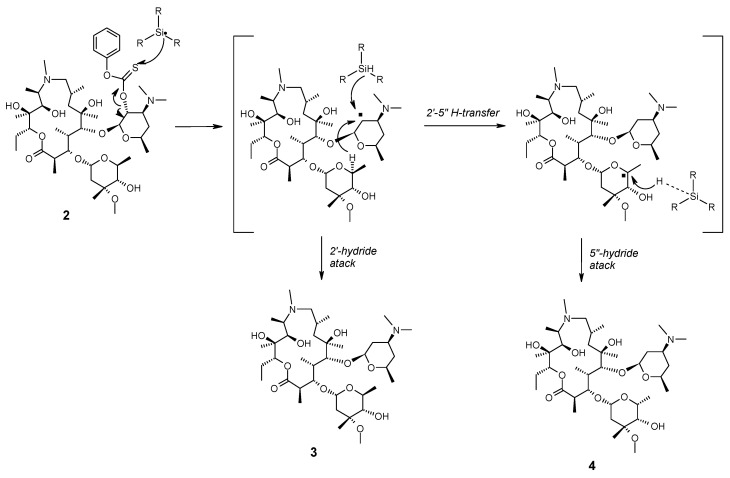
The assumed mechanism of the formation of **3** and **4**.

These results suggested that the possible mechanism of the formation of compound **4** proceeds via 2′-5″ hydrogen transfer and the subsequent attack of the hydride reagent at C-5″ from the less hindered side (Scheme 2).

In order to scale up the synthesis of **4** for biological studies, the reaction sequence was optimized to a one-pot two-step procedure in which *O*-phenyl-thiocarbonate **2** was not isolated prior to the radical deoxygenation reaction since both reactions proceed in toluene as solvent. Sodium hydrogen carbonate was filtered off, the filtrate diluted with fresh toluene, and then radical deoxygenation conditions were applied. The overall yield was around 45%.

### 2.2. Biology

**Figure 2:**
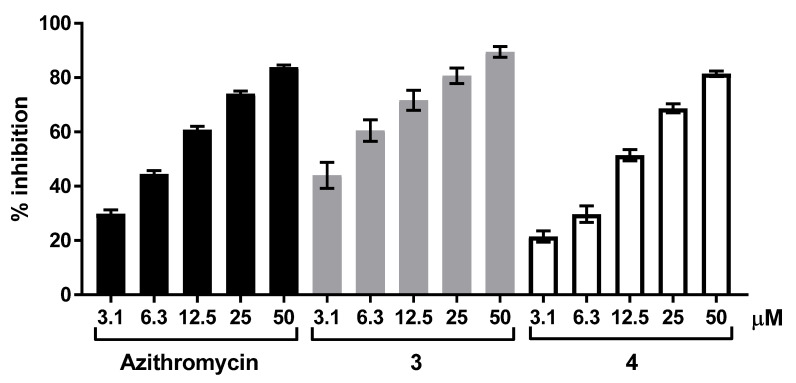
Inhibition of LPS-stimulated IL-6 production in murine splenocytes. Data are presented as means ± S.E.M. of 11 independent experiments for azithromycin and compound **4** and **6** experiments for compound **3**.

Firstly, compounds **3** and **4** were tested for antibacterial and anti-inflammatory activity in vitro. Compound **3** had weak residual antibacterial activity against some of the common respiratory pathogens while compound **4** was antibacterially inactive (Table 3). Both compounds inhibited lipopolysaccharide (LPS)-stimulated IL-6 production in murine splenocytes in vitro with similar strength as azithromycin (Figure 2). The difference in antibiotic activity between **3** and **4** can easily be explained by looking more closely into the mode of macrolide binding to the bacterial ribosome. Macrolides in general show low affinity towards organisms containing guanine at position 2058Ec (such as *Haloarcula marismortui*) compared to those with adenine at the same position [27]. The explanation could be in the ability to form the hydrogen bond between N1 in the nucleotide at position 2058Ec with the macrolide 2′-hydroxyl group [16,17,19]. However, the crystal structures of azithromycin with wild-type *Haloarcula marismortui* (containing guanine, low affinity) [12] and its mutant (containing adenine, high affinity) [17] both show the presence of a hydrogen bond. In other words, the existence of this hydrogen bond alone does not guarantee a high affinity for the ribosome. Therefore, the macrolide’s inability to form this hydrogen bond (such as in the case of compound **3**) does not equal low affinity towards the ribosome. Consequently, by removing the 2′-hydroxy group, we were not able to completely abolish antibacterial activity (**3**).

In epimer **4**, however, the inversion of stereochemistry at C-5″ caused a drastic conformational change of the cladinose sugar and, consequently, the “eastern” side of the molecule. These changes reduced the hydrophobic interactions that the cladinose can establish with the ribosome, reaffirming the importance of this moiety to the ribosomal binding. The overall result is the observed difference between microbial activities of the two epimers. Of course, due to the intact anti-inflammatory activity of epimer **4**, it can be concluded that neither the 2′-hydroxyl group nor cladinose plays an important role in binding to the anti-inflammatory target.

**Figure 3:**
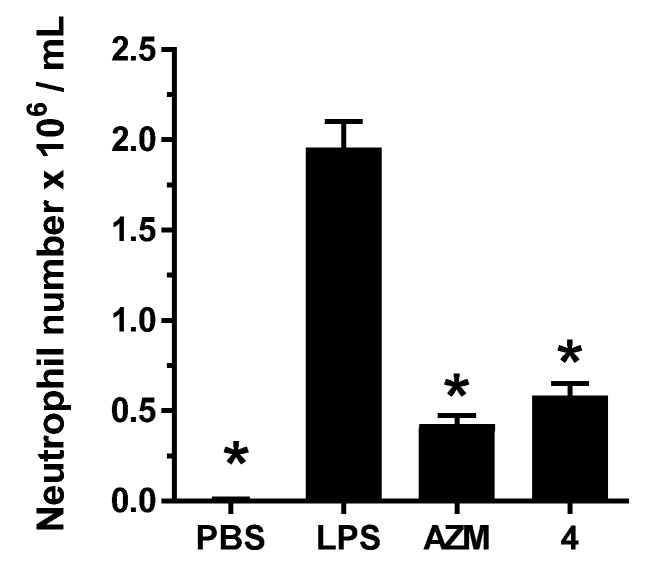
Neutrophil number in BALF 24 h after intranasal LPS challenge of BALB/cJ mice. Compounds were administered at a dose of 200 mg/kg intraperitoneally 2 h prior LPS challenge. Data are presented as means ± S.E.M. of 8 animals per group Asterisk represents *p* < 0.05, one-way ANOVA followed by Dunnett’s multiple comparison test.

**Figure 4:**
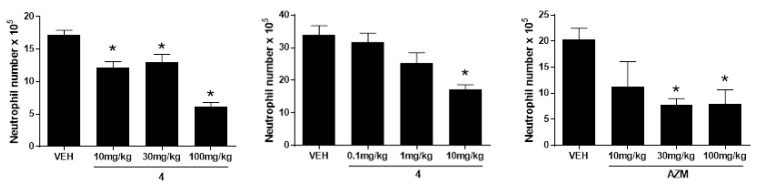
Neutrophil number in BALF 24 h after cigarette-smoke challenge. Compound **4** or azithromycin (AZM) were administered orally 2 h prior smoke challenge on each smoke day. Data are presented as means ± S.E.M. of 5 animals per group. The asterisk represents *p* < 0.05, one-way ANOVA followed by Dunnett’s multiple comparison test.

Since compound **3** had residual antibacterial activity only compound **4** was tested in vivo. The anti-inflammatory activity of compound **4** was compared to the activity of azithromycin in two models of pulmonary inflammation in vivo (Figure 3 and Figure 4). In a murine model of LPS-induced pulmonary inflammation, compound **4** inhibited neutrophil accumulation in bronchoalveolar lavage fluid (BALF) (Figure 3). The activity was comparable to the activity of azithromycin.

The inhibitory activity of compound **4** on lung inflammation was confirmed in the cigarette-smoke-induced pulmonary inflammation model. Similar to azithromycin, the compound dose-dependently inhibited the accumulation of neutrophils in BALF following oral dosing (Figure 4).

To establish if compound **4** could be developed as a drug candidate that could be used orally for long-term treatment and in combination with other therapies, the molecule was tested for cytochrome inhibition. In addition, pharmacokinetic properties in Balb/c mice, Sprague Dawley rats and Beagle dogs were measured. As shown in Table 4, compound **4** had an encouraging pharmacokinetic profile in all species. Overall, compound **4** is characterized by a low systemic clearance (ca. 4.6%, 17.1% and 18% of liver blood flow in mouse, rat, and dog, respectively) and a moderate-to-large volume of distribution in mice and large Vss in rat and dog, indicating extensive distribution into tissues. With a half-life of ca. 11 h in mice, ca. 15 h in rats and ca. 21 h in dogs, compound **4** is suitable for once-daily oral dosing. Finally, compound **4** had a moderate bioavailability in mice (12%) and rats (31%), and a high oral F in dogs (62%). In addition, the compound has a low potential for inducing drug–drug interactions since IC50 values for inhibition of cytochromes 1A2, 2C9, 2C19, 2D6 and 3A4 were higher than 33 µM.

In conclusion, the antibacterial and anti-inflammatory effects of a novel macrolide: 2′-dehydroxy-5″-epi-azithromycin (**4**) were examined. Compound **4** was synthesized using a modified Barton–McCombie radical deoxygenation method. The assumed reaction mechanism involves 2′-5″ hydrogen transfer. Unlike azithromycin and the natural epimer **3,** compound **4** was completely devoid of antibacterial activity. The explanation for this was found in a 3D structural NMR study performed in buffered water, which showed that the inversion of stereochemistry at C-5″ caused a drastic conformational change which diminished the hydrophobic interactions primarily between the cladinose and the ribosome.

However, compound **4** retained anti-inflammatory activity of the parent macrolide, as demonstrated by the inhibition of LPS-stimulated IL-6 production in vitro and reduction of cell accumulation in BALF of LPS- and cigarette-smoke-challenged animals. Therefore, it can be concluded that neither the 2′-hydroxyl group nor cladinose plays an important role in binding to the anti-inflammatory target. Additionally, pharmacokinetic studies revealed that the compound has good oral bioavailability and a half-life that supports once-daily dosing.

## 3. Materials and Methods

### 3.1. Chemistry

Unless noted otherwise, all solvents and chemical reagents were used as supplied. HPLC-MS analysis was performed on Platform LCZ or LCQ Deca instruments. High-resolution mass spectrometry (HRMS) spectra were recorded on Micromass Qtof2 with ESI. For NMR analysis the spectra were acquired using standard Bruker sequences at 25 °C on Bruker Avance DRX500 and Bruker Avance III 600 spectrometers equipped with 5 mm diameter inverse detection probe with z-gradient accessory. The samples were prepared in CDCl_3_ and D_2_O with TMS as the internal standard.

*Azithromycin-2* *′-thiophenylcarbonate (* **2** *):*

To a stirred solution of **1** (10 g, 13.3 mmol) in toluene (200 mL) sodium hydrogen carbonate (16.8 g, 200 mmol) was added. Then *O*-phenyl chlorothionoformate (2 mL, 14.7 mmol) was added in 4 portions (every 20 min). The reaction mixture was heated to 60 °C and stirred for 2 h. Sodium hydrogen carbonate was filtered off and filtrate evaporated. The residue was precipitated from acetonitrile to afford 7.8 g of title product that was used as is in further reactions. MS (ES+) *m*/*z* 885.63 [MH]^+^.

*2* *′-dehydroxy azithromycin (* **3** *):*

To a stirred solution of **2** (1 g, 1.13 mmol) in toluene (10 mL) under argon was added tributyltin hydride (1.59 mL, 5.65 mmol) and AIBN (9.28 mg, 0.056 mmol). The reaction was heated to 90 °C for 3 h and then cooled to RT. The reaction mixture was concentrated in vacuo and the residue purified by Biotage 40 + M using a gradient of 0 to 12% of (10% 0.880 NH_3_ in MeOH)/DCM to afford title product (735 mg, 1.0 mmol, 89 % yield) as a white foam.

HRMS (ES) calc for C_38_H_73_N_2_O_11_ (M+H+) 733.5214, found 733.5208.

^1^H NMR (500 MHz, CDCl_3_) [δ/ppm] 8.98 (6-OH, br. s.), 5.11 (1”-H, d, *J* = 4.7 Hz), 4.68 (13-H, dd, *J* = 10.0, 2.4 Hz), 4.67 (11-OH, br.s), 4.52 (1′-H, dd, *J* = 9.4, 1.7 Hz), 4.28 (3-H, dd, *J* = 4.7, 1.6 Hz), 4.05 (5″-H, dq, *J* = 9.4, 6.2 Hz), 3.66 (11-H, d, *J* = 4.0 Hz), 3.62 (5-H, d, *J* = 7.4 Hz), 3.42 (5′-H, m, *J* = 10.5, 6.2, 1.7 Hz), 3.31 (3”-OCH_3_, s), 3.04 (4”-H, d, *J* = 9.3 Hz), 2.92 (12-OH, s), 2.76 (2-H, dq, *J* = 7.3, 4.9 Hz), 2.69 (10-H, dq, *J* = 6.9, 1.0 Hz), 2.53 (9-H, dd, *J* = 11.5, 1.5 Hz), 2.42 (3′-H, tt, *J* = 11.9, 3.8 Hz), 2.35 (2”-H, d, *J* = 15.3 Hz), 2.32 (9a-CH_3_, s), 2.28 (3′-N(CH_3_)_2_, s), 2.04 (9-H, t, *J* = 11.4 Hz), 2.03 (2′-H, m), 2.02 (8-H, m), 1.93 (4-H, m), 1.89 (14-H, m, *J* = 7.5, 2.3 Hz), 1.75 (7-H, d, *J* = 14.5 Hz), 1.68 (4′-H, m, *J* = 12.5, 1.6 Hz), 1.59 (2”-H, dd, *J* = 15.2, 5.0 Hz), 1.47 (14-H, m, *J* = 14.4, 9.9, 7.2 Hz), 1.35 (6-CH_3_, s), 1.33 (5″-CH_3_, d, *J* = 6.2 Hz), 1.27 (2′-H, ddd, *J* = 12.6, 12.2, 9.5 Hz), 1.25 (3”-CH_3_, s), 1.24 (5′-CH_3_, d, *J* = 6.2 Hz), 1.23 (7-H, dd, *J* = 14.5, 8.3 Hz), 1.19 (2-CH_3_, d, *J* = 7.5 Hz), 1.13 (4′-H, q, *J* = 12.3 Hz), 1.09 (10-CH_3_, d, *J* = 7.0 Hz), 1.08 (12-CH_3_, s), 0.91 (8-CH_3_, d, *J* = 6.7 Hz), 0.90 (4-CH_3_, d, *J* = 8.2 Hz), 0.89 (15-H, t, *J* = 7.0 Hz).

^13^C NMR (125 MHz, CDCl_3_) [δ/ppm] 178.8 (C-1), 101.0 (C-1′), 95.0 (C-1″), 84.6 (C-5), 78.3 (C-3), 78.1 (C-4″), 77.5 (C-13), 74.2 (C-12), 74.0 (C-11), 73.5 (C-6), 73.0 (C-3″), 70.1 (C-9), 68.7 (C-5′), 65.5 (C-5″), 62.3 (C-10), 60.5 (C-3′), 49.3 (3″-OCH_3_), 45.3 (C-2), 42.2 (C-7), 41.8 (C-4), 41.2 (3′-N(CH_3_)_2_), 36.3 (9a-CH_3_), 34.8 (C-2″), 34.3 (C-4′), 33.9 (C-2′), 27.4 (6-CH_3_), 26.7 (C-8), 21.6 (5′-CH_3_), 21.9 (8-CH_3_), 21.6 (3″-CH_3_), 21.2 (C-14), 18.2 (5″-CH_3_), 16.2 (12-CH_3_), 15.0 (2-CH_3_), 11.2 (C-15), 9.0 (4-CH_3_), 7.4 (10-CH_3_).

*2′-dehydroxy-5″-epi-azithromycin (* **4** *):*

To a stirred solution of **1** (10 g, 13.3 mmol) in toluene (200 mL) sodium hydrogen carbonate (16.8 g, 200 mmol) was added. Then *O*-phenyl chlorothionoformate (2 mL, 14.7 mmol) was added in 4 portions (every 20 min). The reaction mixture was heated to 60 ^o^C and stirred for 2 h. Sodium hydrogen carbonate was filtered off and filtrate diluted with fresh toluene (180 mL). Toluene solution was purged with nitrogen (1 h), ACCN (1.63 g, 0.67 mmol) was added, and the reaction mixture heated to 115 °C. Then tris(trimethylsilyl)silane (4.95 mL, 16.0 mmol) and stirring at 115 °C continued for 2 h. Toluene was evaporated, the residue dissolved in water (200 mL)/ethyl acetate (100 mL) mixture, and pH adjusted to 5.5. The layers were separated and extraction with ethyl acetate repeated one more time at pH 5.5 and two times at pH 6.5. The water layer was then extracted with dichloromethane at pH 9.7. Dichloromethane layer was dried over anhydrous Na_2_SO_4_ and evaporated. The residue was precipitated from acetonitrile to afford 4.3 g of title product as a white foam.

HRMS (ES) calc for C_38_H_73_N_2_O_11_ (M+H+) 733.5214, found 733.5211.

^1^H NMR (600 MHz, CDCl_3_) [δ/ppm] 7.16 (6-OH, br. s.), 4.76 (1′-H, dd, *J* = 9.1, 1.0 Hz), 4.61 (13-H, d, *J* = 10.5 Hz), 4.47 (1”-H, d, *J* = 9.4 Hz), 4.07 (5-H, d, *J* = 3.1 Hz), 3.89 (3-H, d, *J* = 10.1 Hz), 3.68 (5′-H, qd, *J* = 6.2, 2.0 Hz), 3.67 (11-OH, d, *J* = 5.9 Hz), 3.58 (11-H, d, *J* = 5.9 Hz), 3.50 (5″-H, q, *J* = 6.3 Hz), 3.23 (3”-OCH_3_, s), 3.19 (4”-H, s), 2.89 (2-H, dq, *J* = 10.1, 7.0 Hz), 2.70 (10-H, q, *J* = 6.5 Hz), 2.63 (4”-OH, s), 2.61 (12-OH, s), 2.53 (3′-H, tt, *J* = 12.0, 3.6 Hz), 2.45 (9-H, dd, *J* = 12.2, 2.8 Hz), 2.33 (9a-CH_3_, s), 2.24 (3′-N(CH_3_)_2_, s), 2.04 (4-H, m), 2.01 (9-H, t, *J* = 11.9 Hz), 1.98 (2′-H, m), 1.89 (8-H, m), 1.87 (14-H, dq, *J* = 14.3, 7.3 Hz), 1.80 (2”-H, d, *J* = 12.2 Hz), 1.70 (7-H, d, *J* = 14.3 Hz), 1.63 (2”-H, dd, *J* = 12.2, 9.4 Hz), 1.60 (4′-H, br. s., *J* = 11.5, 4.0, 1.8 Hz), 1.52 (14-H, m, *J* = 14.3, 10.8, 7.3, 7.3, 7.3 Hz), 1.36 (5″-CH_3_, d, *J* = 6.3 Hz), 1.30 (6-CH_3_, s), 1.21 (5′-CH_3_, d, *J* = 5.9 Hz), 1.20 (3”-CH_3_, s), 1.20 (2-CH_3_, d, *J* = 7.0 Hz), 1.16 (2′-H, q, *J* = 11.5 Hz), 1.15 (7-H, dd, *J* = 14.0, 8.0 Hz), 1.10 (4′-H, q, *J* = 11.5 Hz), 1.07 (10-CH_3_, d, *J* = 7.0 Hz), 1.02 (12-CH_3_, s), 0.87 (8-CH_3_, d, *J* = 7.3 Hz), 0.86 (15-H, t, *J* = 7.7 Hz), 0.86 (4-CH_3_, d, *J* = 7.7 Hz).

^13^C NMR (125 MHz, CDCl_3_) [δ/ppm] 178.1 (C-1), 102.1 (C-1′), 100.2 (C-1″), 87.3 (C-5), 83.3 (C-3), 78.2 (C-13), 76.2 (C-11), 75.7 (C-3″), 74.4 (C-12), 73.6 (C-6), 72.1 (C-4″), 71.1 (C-9), 70.0 (C-5″), 68.7 (C-5′), 62.3 (C-10), 60.3 (C-3′), 48.7 (3″-OCH_3_), 44.4 (C-2), 41.6 (C-7), 41.4 (3′-N(CH_3_)_2_), 37.3 (C-4), 37.1 (C-2″), 36.9 (9a-CH_3_), 35.0 (C-4′), 33.8 (C-2′), 26.6 (C-8), 26.4 (6-CH_3_), 21.9 (5′-CH_3_), 21.7 (8-CH_3_), 20.9 (C-14), 19.8 (3″-CH_3_), 17.1 (5″-CH_3_), 16.3 (2-CH_3_), 16.2 (12-CH_3_), 11.3 (C-15), 9.7 (4-CH_3_), 7.6 (10-CH_3_).

### 3.2. Biology

#### 3.2.1. Antimicrobial Activity

The Clinical and Laboratory Standards (CLSI) recommended procedure (Document M7-A6A7, Methods for Dilution Susceptibility Tests for Bacteria that Grow Aerobically) by broth microdilution was used to determine the antimicrobial activity of compounds against *Escherichia coli* (ATCC25922), *Staphylococcus aureus* (ATCC13709), *Streptococcus pneumoniae* (ATCC49619), *Streptococcus pyogenes* (ATCC700294), *Moraxella catarrhalis* (ATCC23246) and *Haemophilus influenzae* (ATCC49247). The minimum inhibitory concentration (MIC) is determined as the lowest concentration of compound that inhibited visible growth.

#### 3.2.2. LPS-Induced IL-6 Production by Murine Splenocytes

Splenocytes were isolated from spleens of BALB/cJ mice (Charles River, France) resuspended in DMEM supplemented with 1% FBS and seeded in a 24-well plate. Cells were pre-incubated with the test compounds for 2 h, stimulated with 1 μg/mL LPS from *E. coli* 0111:B4 (Sigma Chemical Corp. Saint Louis, USA) and incubated overnight. Concentration of IL-6 was determined in cell supernatants by sandwich ELISA using capture and detection antibodies (R&D Systems, Minneapolis, USA) according to the manufacturer’s recommendations.

#### 3.2.3. LPS-Induced Pulmonary Neutrophilia

Vehicle, azithromycin, and compound **4** were administered intraperitoneally at a dose of 200 mg/kg (b.w.) to 10-week-old male BALB/cJ mice (Charles River, Lyon, France) 2 h before intranasal challenge with 2 μg of LPS from *E. coli*. Then, 24 h after LPS application animals were euthanized and bronchoalveolar lavage was performed. Total number of cells in BALF was counted with a hematological analyzer (Sysmex SF 3000, Sysmex Corp., Kobe, Japan). Percentages of neutrophils were determined by morphological examination of at least 200 cells on smears prepared by cytocentrifugation (Cytospin-3, Thermo Fisher Scientific Inc, Waltham, MA, USA) and stained with Kwik-Diff staining set (Thermo Fisher Scientific Inc., USA).

#### 3.2.4. Cigarette-Smoke-Induced Pulmonary Inflammation

Compounds were dosed by oral gavage, 2 h prior to the first cigarette each day of the smoke challenge. Then, 2 h after compound administration, mice were placed into a plexiglass box (Braintree Scientific, size 10” ×4” ×4”). Cigarette smoke was introduced into the box via a peristaltic pump (Masterflex L/S, Digital Economy Drive) set at 40 mL/min. Breathing air was also introduced at a rate of 0.4 L/min. A total of three cigarettes (research type 4A1, University of Kentucky Tobacco Institute) were given back-to-back with one-minute period between each one when the mice breathed fresh air for one minute. Then, 2 h after the first group of cigarettes, two additional cigarettes were given, again allowing 1 min between each cigarette. This procedure was performed for 2 days. On the third day, mice were euthanized and bronchoalveolar lavage was performed. Slides made on a cytospin were stained with Kwik-Diff for differential cell counts. Total cell counts were performed with a hemocytometer.

#### 3.2.5. Statistical Analysis

All values are presented as means ± S.E.M. To define statistically significant differences in cell numbers among vehicle-treated and macrolide-treated mice the data were subjected to one-way ANOVA followed by Dunnett’s Multiple Comparison Test using GraphPad Prism version 5.00 for Windows (GraphPad Software, San Diego, CA, USA). The level of significance was set at *p* < 0.05 in all cases.

#### 3.2.6. In Vitro Cytochrome Inhibition

Test compounds were incubated using a concentration range from 0.1 to 100 µM with recombinant human CYP1A2, 2C9, 2C19, 2D6 and 3A4 (Cypex). The compounds were preincubated at 37 °C for 5 or 10 min with the CYP enzyme isoforms, in the presence of 50 mM phosphate buffer (pH 7.4) and fluorescent probes. The incubations were started by addition of a cofactor solution containing NADP, glucose 6-phosphate and glucose 6-phosphate dehydrogenase. The plates were incubated for 10 min at 37 °C reading the fluorescence signal on a plate reader throughout. The fluorescence data were then used to calculate the IC_50_ values for each compound.

#### 3.2.7. Pharmacokinetics

Pharmacokinetic profiles were determined in male Balb/c mice and CD Sprague-Dawley rats (Charles River Laboratories, Germany), following single oral (p.o.) and single intravenous (i.v.) bolus administration. The compound was administered at a dose of 2 mg/mg i.v. and 10 mg/kg p.o. Blood was obtained by serial blood sampling from the tail vein over a range of time points up to 30 h postdose. Samples were extracted by protein precipitation and subjected to quantitative analysis by LC-MS/MS using compound-specific mass transitions. Drug concentration−time profiles were generated and noncompartmental PK analysis used to generate estimates of half-life, clearance, volume of distribution, and oral bioavailability.

## Figures and Tables

**Table 1:** Radical deoxygenation conditions (toluene was used as solvent in all reactions).

Radical Quench/Eq.	Radical Initiator/Eq.	Concentration (Mol/L)	Temperature (°C)	Ratio of 3:4
Bu_3_SnH/1.2	AIBN/0.05	0.056	90	2.5:1
Bu_3_SnH/5	AIBN/0.05	0.11	90	1:0
(TMS)_3_SiH/1.2	AIBN/0.05	0.056	90	1:2
(TMS)_3_SiH/1.2	AIBN/0.05	0.023	90	1:3
(TMS)_3_SiH/1.2	ACCN/0.05	0.056	90	1:4
(TMS)_3_SiH/1.1	ACCN/0.5	0.035	115	1:12

**Table 2:** Comparison of ^3^J_H,H_ (Hz) coupling constants for **3** and **4** in D_2_O at 25 °C; differences marked in bold.

Atom	Cpd 3	Cpd 4	Atom	Cpd 3	Cpd 4
**2**, **3**	**7.0**	**11.0**	2, 2 CH_3_	7.7	7.5
3, 4	1.8	2.0	1″, 2″a	<1	<1
**4**, **5**	**8.0**	**2.6**	**1″**, **2″b**	**4.9**	**11.1**
7a, 8	<1	<1	**4″**, **5″**	**9.8**	**<1**
7b, 8	8.2	9.0	4, 4 CH_3_	7.7	7.7
8, 9a	2.1	3.2	8, 8 CH_3_	7.2	7.2
8, 9b	13.0	12.6	10, 10 CH_3_	7.2	7.2
10, 11	<1	1.0	14, 15	7.7	7.6
13, 14a	2.3	2.3	5′, 5′ CH_3_	6.2	6.4
13, 14b	10.8	11.1	5″, 5″ CH_3_	6.4	6.9
1′, 2′a	2.0	1.9			
1′, 2′b	9.5	9.8	7a, 7b	15.9	17.2
2′a, 3′	4.0	4.0	9a, 9b	13.0	12.6
2′b, 3′	13.1	13.1	14a, 14b	14.6	14.6
3′, 4′a	4.0	4.0	2′a, 2′b	13.1	12.6
3′, 4′b	12.8	*	4′a, 4′b	13.0	12.8
4′a, 5′	1.8	1.8	**2″a**, **2″b**	**16.4**	**12.6**
4′b, 5′	11.3	11.3			

**Table 3:** Antibacterial activity (expressed as MIC in μg/mL) against *S. aureus*, *S. pneumoniae*, *S. pyogenes*, *M. catarrhalis*, *H. influenzae*, and *E. coli*.

Compounds	*S. aureus*	*S. penumoniae*	*S. pyogenes*	*M. catarrhalis*	*H. influenzae*	*E. coli*
AZM	0.5	<0.125	<0.125	<0.125	2	2
**3**	>64	32	64	16	>64	>64
**4**	>64	>64	>64	>64	>64	>64

**Table 4:** Pharmacokinetic profile of compound **4** in mouse, rat and dog.

	CL	Vss	t _1/2_	Oral F
	(mL/min/kg)	(L/kg)	(h)	(%)
mouse	4.1 ± 1.1	2 ± 0.8	10.5 ± 2.8	12.4 ± 4.2
rat	14.5 ± 8.5	11.7 ± 1.9	15.4 ± 6.1	27 ± 6
dog	5.5 ± 0.9	6.9 ± 2.0	20.5 ± 3.5	62 ± 10

## Data Availability

Not Applicable.

## Supplementary Materials

The following are available online, Figure S1. 1H spectrum, structure, numbering and assignment of 3 in D2O at 25 °C; Figure S2. 13C spectrum, structure, numbering and assignment of 3 in D2O at 25 °C; Figure S3. ROESY spectrum of 3 in D2O at 25 °C; Figure S4. 1H spectrum, structure, numbering and assignment of 4 in D2O at 25 °C; Figure S5. 13C spectrum, structure, numbering and assignment of 4 in D2O at 25 °C; Figure S3. ROESY spectrum of 4 in D2O at 25 °C; Figure S7. 1H spectrum of 4 in CDCl3 at 25 °C; Figure S8. 13C spectrum of 4 in CDCl3 at 25 °C; Figure S9. 1H-1H COSY spectrum of 4 in CDCl3 at 25 °C; Figure S10. 1H-13C HSQCed spectrum of 4 in CDCl3 at 25 °C: Figure S11. 1H-13C HMBC spectrum of 4 in CDCl3 at 25 °C; Figure S12. Full 1H spectrum of 3 in D2O at 25 °C; Figure S13. Full 1H spectrum of 4 in D2O at 25 °C; Table S1. Comparison of nOe interactions (from ROESY) for 3 and 4 in D2O at 25 °C.
