# Supplementary material for: Unprecedented Epimerization of an Azithromycin Analogue: Synthesis, Structure and Biological Activity of 2′-Dehydroxy-5″-Epi-Azithromycin
Source: Molecules. 2022 Feb 3;27(3):1034. doi: 10.3390/molecules27031034 (PMC8838534; doi:10.3390/molecules27031034)
Supplement: Supplementary file 1 [file molecules-27-01034-s001.zip › molecules-1536977-suppl.pdf]

Supplementary Material

Novel anti-inflammatory macrolide without antimicrobial activity: 2′-dehydroxy-5″-epi-azithromycin

Goran Kragol, Victoria A. Steadman, Zorica Marušić Ištuk, Ana Čikoš, Martina Bosnar, Dubravko Jelić, Gabrijela Ergović, Marija Trzun, Berislav Bošnjak, Ana Bokulić, Jasna Padovan, Ines Glojnarić and Vesna Eraković Haber

Figure S1. ^{1}H spectrum, structure, numbering and assignment of **3** in D_2O at 25 °C................................ 2
Figure S2. ^{13}C spectrum, structure, numbering and assignment of **3** in D_2O at 25 °C............................... 3
Figure S3. ROESY spectrum of **3** in D_2O at 25 °C 4
Figure S4. ^{1}H spectrum, structure, numbering and assignment of **4** in D_2O at 25 °C................................ 5
Figure S5. ^{13}C spectrum, structure, numbering and assignment of **4** in D_2O at 25 °C............................... 6
Figure S3. ROESY spectrum of **4** in D_2O at 25 °C 7
Figure S7. ^{1}H spectrum of **4** in $CDCl_3$ at 25 °C 8
Figure S8. ^{13}C spectrum of **4** in $CDCl_3$ at 25 °C 9
Figure S9. ^{1}H-^{1}H COSY spectrum of **4** in $CDCl_3$ at 25 °C 10
Figure S10. ^{1}H-^{13}C HSQCed spectrum of **4** in $CDCl_3$ at 25 °C 11
Figure S11. ^{1}H-^{13}C HMBC spectrum of **4** in $CDCl_3$ at 25 °C 12
Figure S12. Full ^{1}H spectrum of **3** in D_2O at 25 °C 13
Figure S13. Full ^{1}H spectrum of **4** in D_2O at 25 °C 14

Table S1. Comparison of nOe interactions (from ROESY) for **3** and **4** in D_2O at 25 °C; differences marked in red, overlap of 4'b, 5' CH_3 and 5'' CH_3 marked in yellow 15

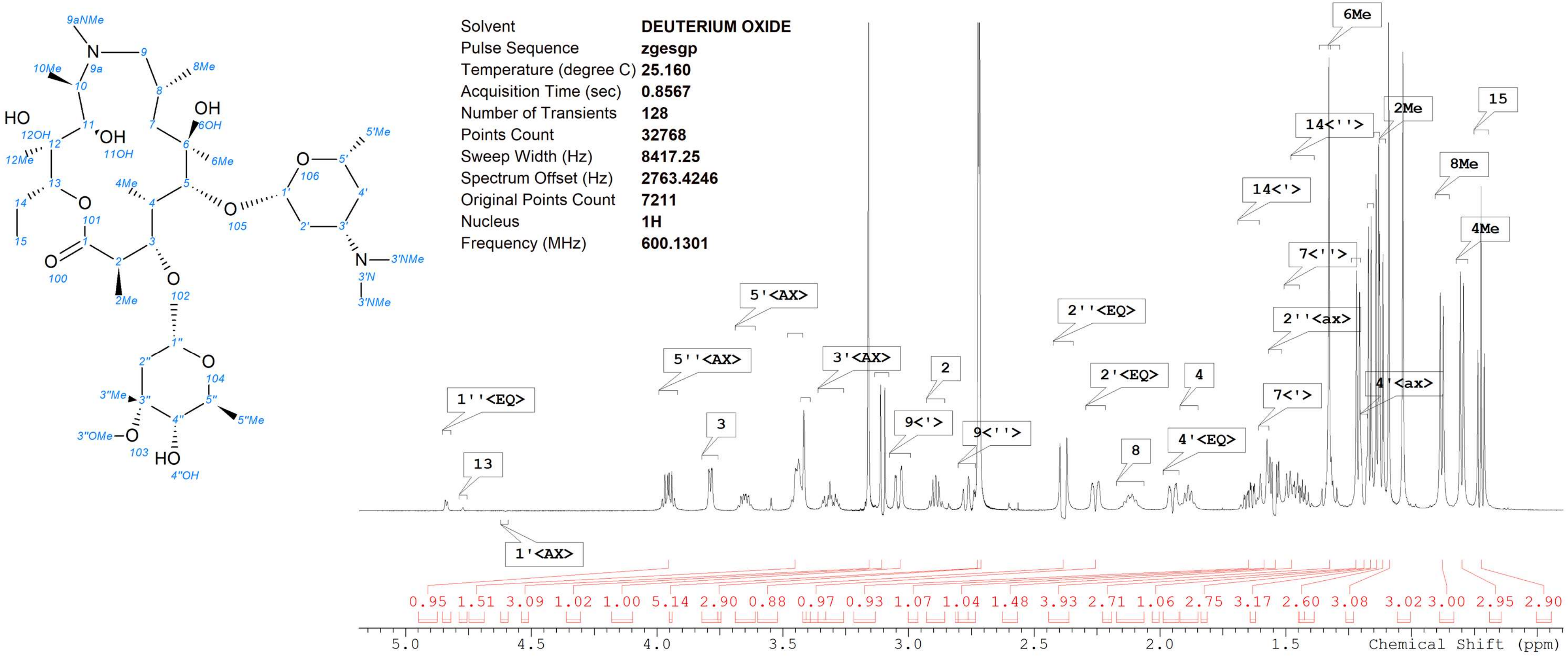

Shift1 (ppm)	H's	Type	J (Hz)	Atom1	Multiplet1
0.724	3.000	t	7.7	15	M01
0.800	3.000	d	7.7	4Me	M02
0.880	3.000	d	7.2	8Me	M03
1.035	3.000	s	-	12Me	M04
1.091	3.000	s	-	3"Me	M05
1.120	3.000	d	7.7	2Me	M06
1.136	3.000	d	6.2	5'Me	M07
1.166	3.000	d	6.4	5"Me	M08
1.189	1.000	q	13.0, 12.0, 11.3	4'<ax>	M09
1.213	3.000	d	7.2	10Me	M10
1.327	1.000	td	13.1, 13.0, 9.5	2'<ax>	M12
1.329	3.000	s	-	6Me	M11
1.438	1.000	m	14.6, 10.8, 7.4	14<">	M13
1.477	1.000	br dd	15.9, 8.2	7<">	M14
1.546	1.000	dd	16.2, 4.9	2"<ax>	M15

Shift1 (ppm)	H's	Type	J (Hz)	Atom1	Multiplet1
1.588	1.000	br d	15.7	7<'>	M16
1.645	1.000	m	14.6, 8.0, 2.3	14<'>	M17
1.888	1.000	quin	8.2	4	M18
1.950	1.000	dt	13.0, 4.0, 2.0	4'<EQ>	M19
2.119	1.000	m	13.0, 8.2, 7.2, 1.6	8	M20
2.257	1.000	dt	13.0, 4.0, 2.0	2'<EQ>	M23
2.385	1.000	d	16.4	2"<EQ>	M24
2.718	3.000	s	-	9aNMe	M25
2.724	6.000	s	-	3'NMe, 3'NMe	M26
2.762	1.000	t	12.8	9<">	M22
2.892	1.000	quin	7.7, 7.0	2	M27
3.041	1.000	dd	13.1, 2.1	9<'>	M21
3.103	1.000	d	9.8	4"<AX>	M28
3.160	3.000	s	-	3"OMe	M29
3.313	1.000	tt	13.1, 12.8, 4.0	3'<AX>	M30

Shift1 (ppm)	H's	Type	J (Hz)	Atom1	Multiplet1
3.418	1.000	s	-	11	M32
3.450	2.000	m	-	5, 10	M33
3.652	1.000	m	11.3, 6.4, 1.8	5'<AX>	M31
3.788	1.000	dd	6.2, 1.8	3	M34
3.956	1.000	dq	10.0, 6.4	5"<AX>	M35
4.614	1.000	s	-	1'<AX>	M36
4.773	1.000	d	1.8	13	M37
4.839	1.000	d	4.6	1"<EQ>	M38

***Figure S1**. ^{1}H spectrum, structure, numbering and assignment of **3** in D_2O at 25 °C*

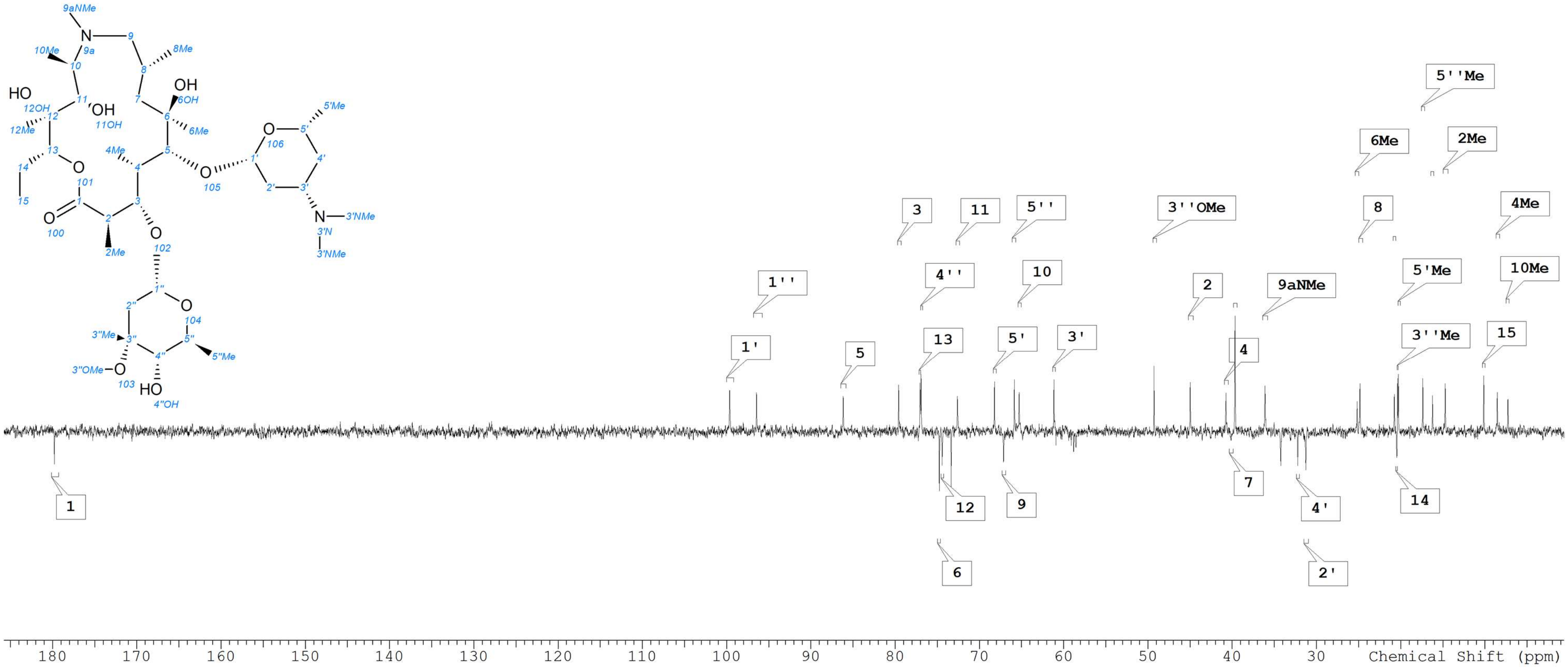

Shift1 (ppm)	C's	Type	Atom1	Multiplet1
7.294	1.000	s	10Me	M37
8.562	1.000	s	4Me	M36
10.164	2.000	s	15	M35
14.747	1.000	s	2Me	M34
16.246	1.000	s	12Me	M33
17.395	2.000	s	5''Me	M32
20.289	2.000	s	5'Me	M31
20.392	2.000	s	3''Me	M30
20.480	1.000	s	14	M29
20.773	1.000	s	8Me	M28
24.856	1.000	s	8	M27
25.189	1.000	s	6Me	M26
31.279	1.000	s	2'	M25
32.230	1.000	s	4'	M24
34.260	1.000	s	2''	M23

Shift1 (ppm)	C's	Type	Atom1	Multiplet1
36.124	1.000	s	9aNMe	M22
39.684	3.000	s	3'NMe, 3'NMe	M21
40.144	1.000	br s	7	M20
40.762	1.000	s	4	M19
44.996	1.000	s	2	M18
49.294	2.000	s	3''OMe	M17
61.171	1.000	s	3'	M16
65.302	1.000	s	10	M15
65.881	1.000	s	5''	M14
67.158	1.000	s	9	M13
68.228	1.000	s	5'	M12
72.629	1.000	s	11	M08
73.366	1.000	s	3''	M11
74.444	1.000	s	12	M10
74.777	1.000	s	6	M09

Shift1 (ppm)	C's	Type	Atom1	Multiplet1
76.942	2.000	s	4''	M07
77.061	1.000	s	13	M06
79.606	1.000	s	3	M05
86.187	1.000	s	5	M04
96.471	1.000	s	1''	M03
99.659	1.000	s	1'	M02
179.805	1.000	s	1	M01

Solvent	**DEUTERIUM OXIDE**
Pulse Sequence	**jmod**
Temperature (degree C)	**25.160**
Acquisition Time (sec)	**1.6712**
Number of Transients	**26127**
Points Count	**32768**
Sweep Width (Hz)	**19607.25**
Spectrum Offset (Hz)	**9027.4482**
Original Points Count	**32768**
Nucleus	**13C**
Frequency (MHz)	**75.4678**

Figure S2. *^{13}C spectrum, structure, numbering and assignment of **3** in D_2O at 25 °C*

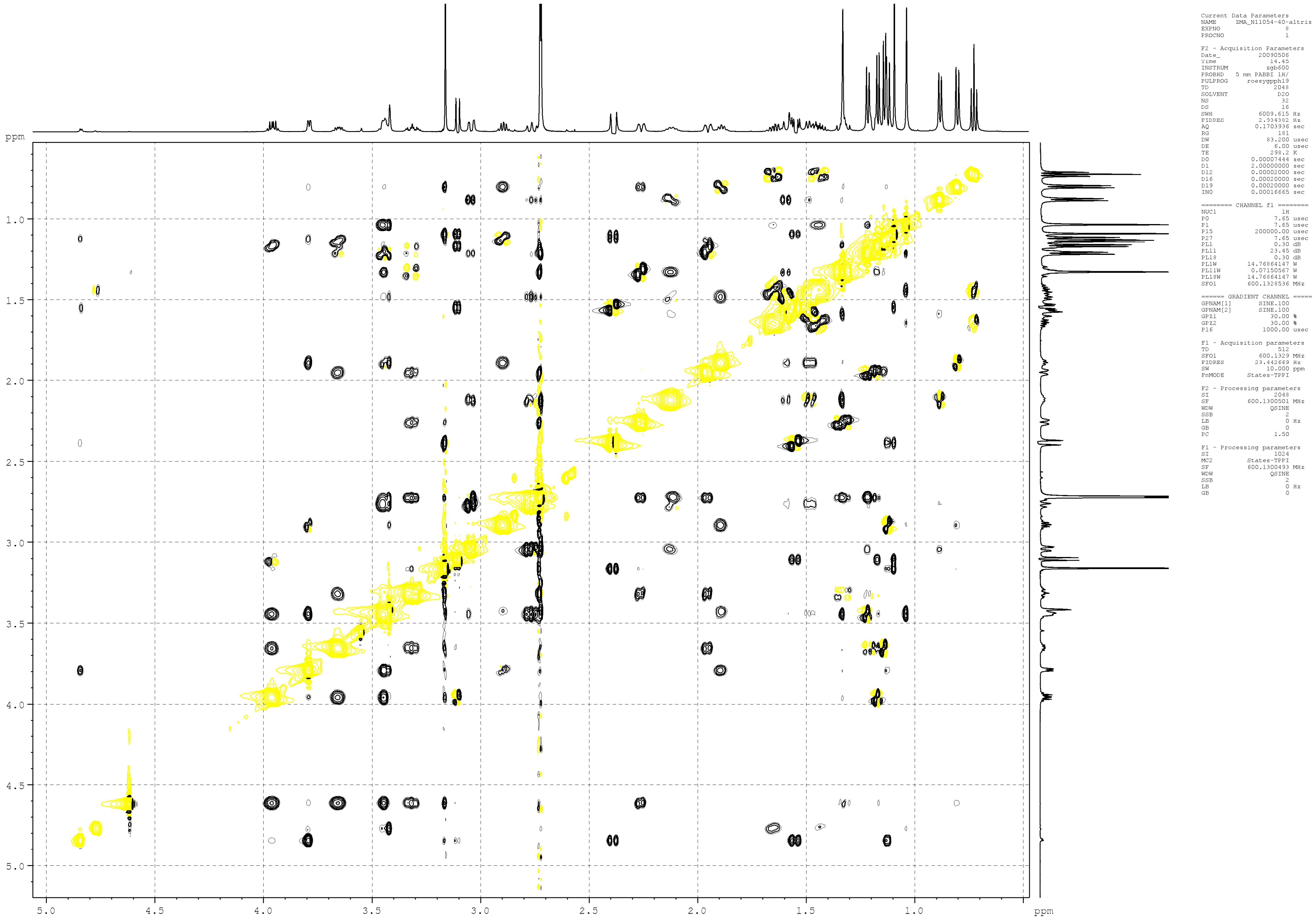

Figure S3. *ROESY spectrum of **3** in D_2O at 25 °C*

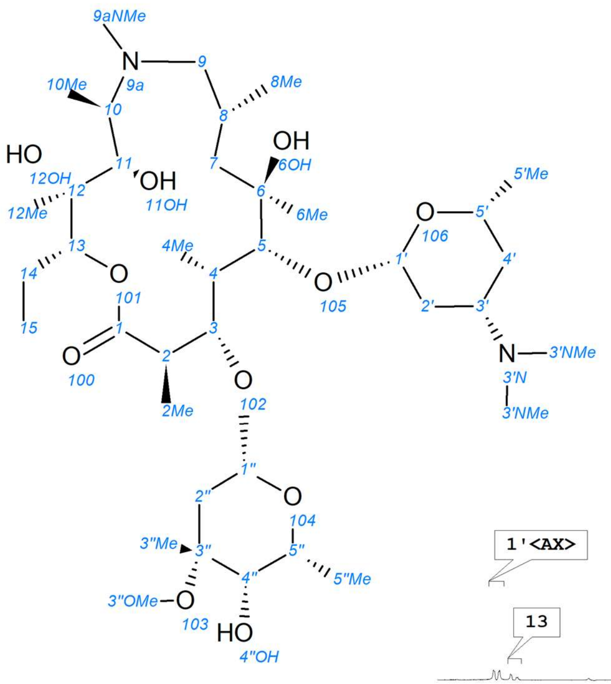

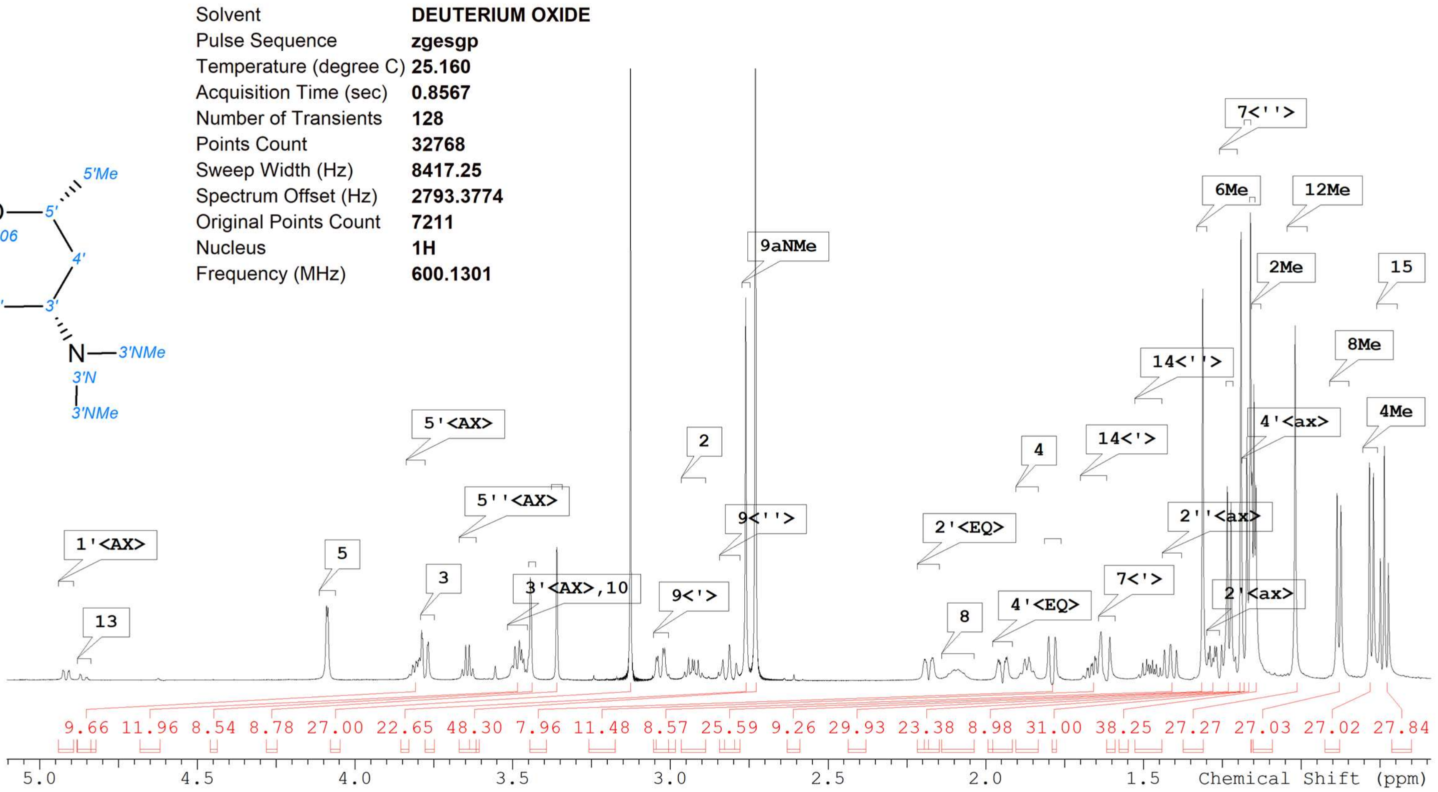

Shift1 (ppm)	H's	Type	J (Hz)	Atom1	Multiplet1
0.737	3.000	t	7.6	15	M01
0.778	3.000	d	7.7	4Me	M02
0.881	3.000	d	7.2	8Me	M03
1.019	3.000	s	-	12Me	M04
1.150	3.000	br d	7.4	2Me	M06
1.156	3.000	br d	6.4	5'Me	M05
1.167	3.000	br d	6.9	5"Me	M07
1.181	1.000	m	-	4'<ax>	M09
1.192	3.000	s	-	3"Me	M08
1.229	3.000	d	7.2	10Me	M10
1.231	1.000	br dd	17.2, 9.0	7<">	M11
1.282	1.000	br dd	12.6, 3.1	2'<ax>	M12
1.313	3.000	s	-	6Me	M13
1.415	1.000	br dd	12.0, 11.0	2"<ax>	M14
1.475	1.000	m	14.6, 11.0, 7.4	14<">	M15
1.622	1.000	br d	17.2	7<'>	M16
1.658	1.000	m	14.6, 7.7, 2.3	14<'>	M17
1.791	1.000	br d	12.6	2"<EQ>	M18
1.870	1.000	qt	7.7, 2.6, 2.0	4	M19
1.949	1.000	dt	12.8, 4.0, 2.1	4'<EQ>	M20
2.094	1.000	m	12.6, 9.0, 7.2, 3.2	8	M21
2.182	1.000	dt	12.4, 4.0, 1.9	2'<EQ>	M22
2.731	6.000	s	-	3'NMe, 3'NMe	M23
2.762	3.000	s	-	9aNMe	M24
2.813	1.000	br t	12.6	9<">	M25
2.928	1.000	dq	11.0, 7.4	2	M26
3.032	1.000	br dd	13.0, 3.2	9<'>	M27
3.127	4.000	s	-	3"OMe	M28
3.361	1.000	s	-	4"<EQ>	M29
3.444	1.000	d	1.3	11	M30
3.478	2.000	m	-	3'<AX>, 10	M31
3.644	1.000	q	6.5	5"<AX>	M32
3.779	1.000	dd	11.0, 1.5	3	M33
3.802	1.000	m	11.3, 6.7, 1.8	5'<AX>	M34
4.089	1.000	d	2.6	5	M35
4.861	1.000	m	11.6, 2.3	13	M37
4.917	1.000	dd	9.8, 1.8	1'<AX>	M36

Figure S4. *^{1}H spectrum, structure, numbering and assignment of **4** in D_2O at 25 °C*

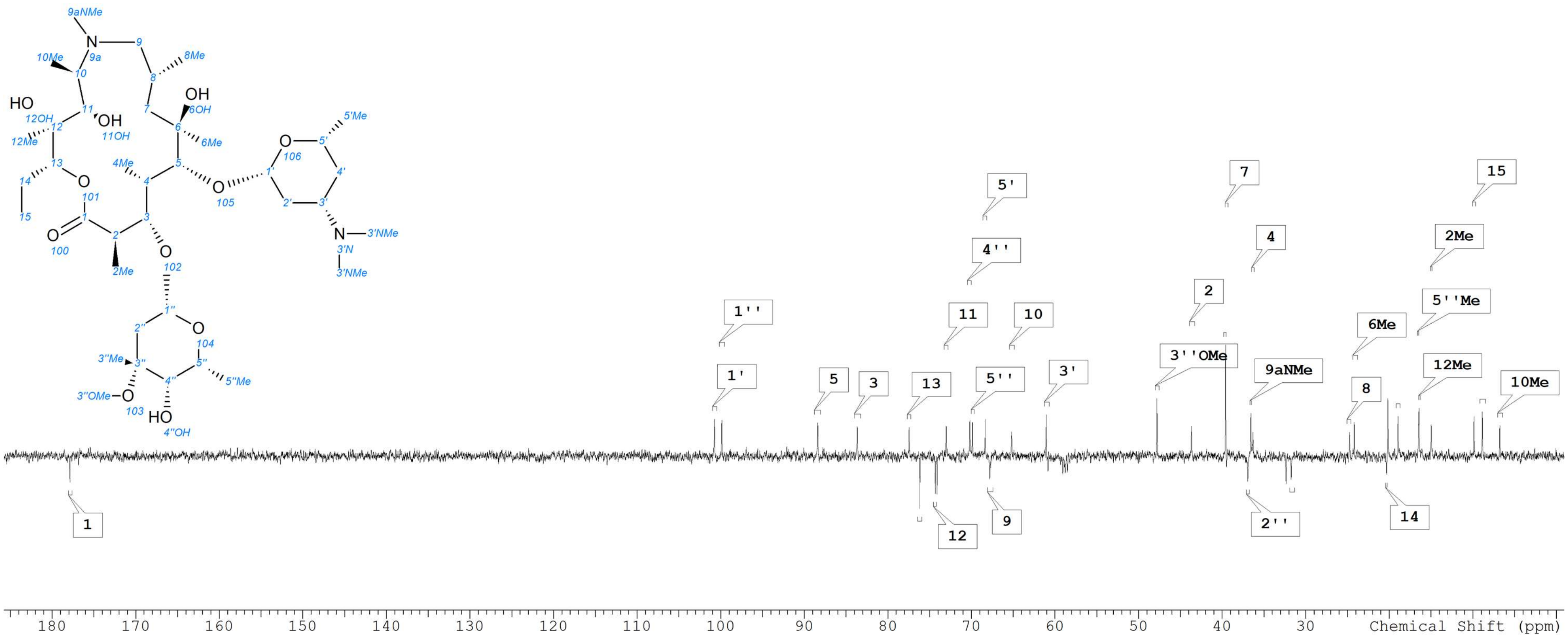

Shift1 (ppm)	C's	Type	Atom1	Multiplet1
6.794	1.000	s	10Me	M36
8.887	2.000	s	4Me	M35
9.879	2.000	s	15	M34
15.001	1.000	s	2Me	M33
16.468	2.000	br s	12Me	M32
16.507	1.000	br s	5''Me	M31
18.973	2.000	s	3''Me	M30
20.186	3.000	s	8Me, 5'Me	M29
20.337	1.000	s	14	M28
24.214	1.000	s	6Me	M27
24.761	1.000	s	8	M26
31.755	1.000	s	2'	M25
32.365	1.000	s	4'	M24
36.330	1.000	s	4	M23
36.576	2.000	s	9aNMe	M22

Shift1 (ppm)	C's	Type	Atom1	Multiplet1
36.940	1.000	s	2''	M21
39.501	5.000	s	7	M20
39.604	4.000	s	3'NMe, 3'NMe	M19
43.688	1.000	s	2	M18
47.811	2.000	s	3''OMe	M17
61.068	2.000	s	3'	M16
65.207	1.000	s	10	M15
67.816	1.000	br s	9	M14
68.379	1.000	s	5'	M13
69.901	1.000	s	5''	M12
70.210	1.000	s	4''	M11
73.041	1.000	s	11	M10
74.127	1.000	s	6	M09
74.333	1.000	s	12	M08
76.189	2.000	s	3''	M07

Shift1 (ppm)	C's	Type	Atom1	Multiplet1
77.473	1.000	s	13	M06
83.682	1.000	s	3	M05
88.407	1.000	s	5	M04
99.889	1.000	s	1''	M03
100.745	1.000	s	1'	M02
177.894	1.000	s	1	M01

Solvent	**DEUTERIUM OXIDE**
Pulse Sequence	**jmod**
Temperature (degree C)	**25.160**
Acquisition Time (sec)	**1.6712**
Points Count	**32768**
Sweep Width (Hz)	**19607.25**
Spectrum Offset (Hz)	**9027.4482**
Original Points Count	**32768**
Nucleus	**13C**
Frequency (MHz)	**75.4678**

Figure S5. *^{13}C spectrum, structure, numbering and assignment of **4** in D_2O at 25 °C*

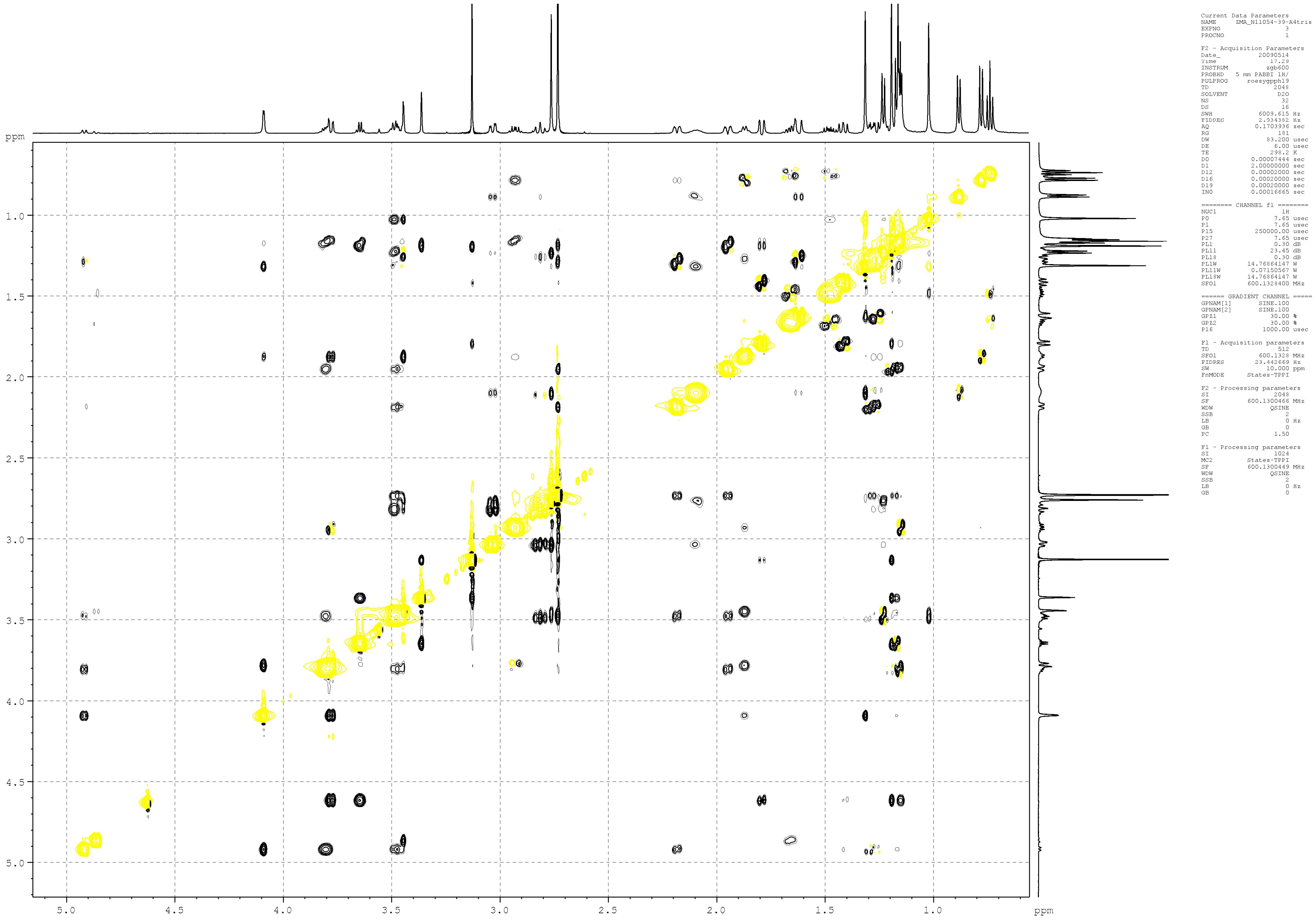

Figure S6. *ROESY spectrum of **4** in D_2O at 25 °C*

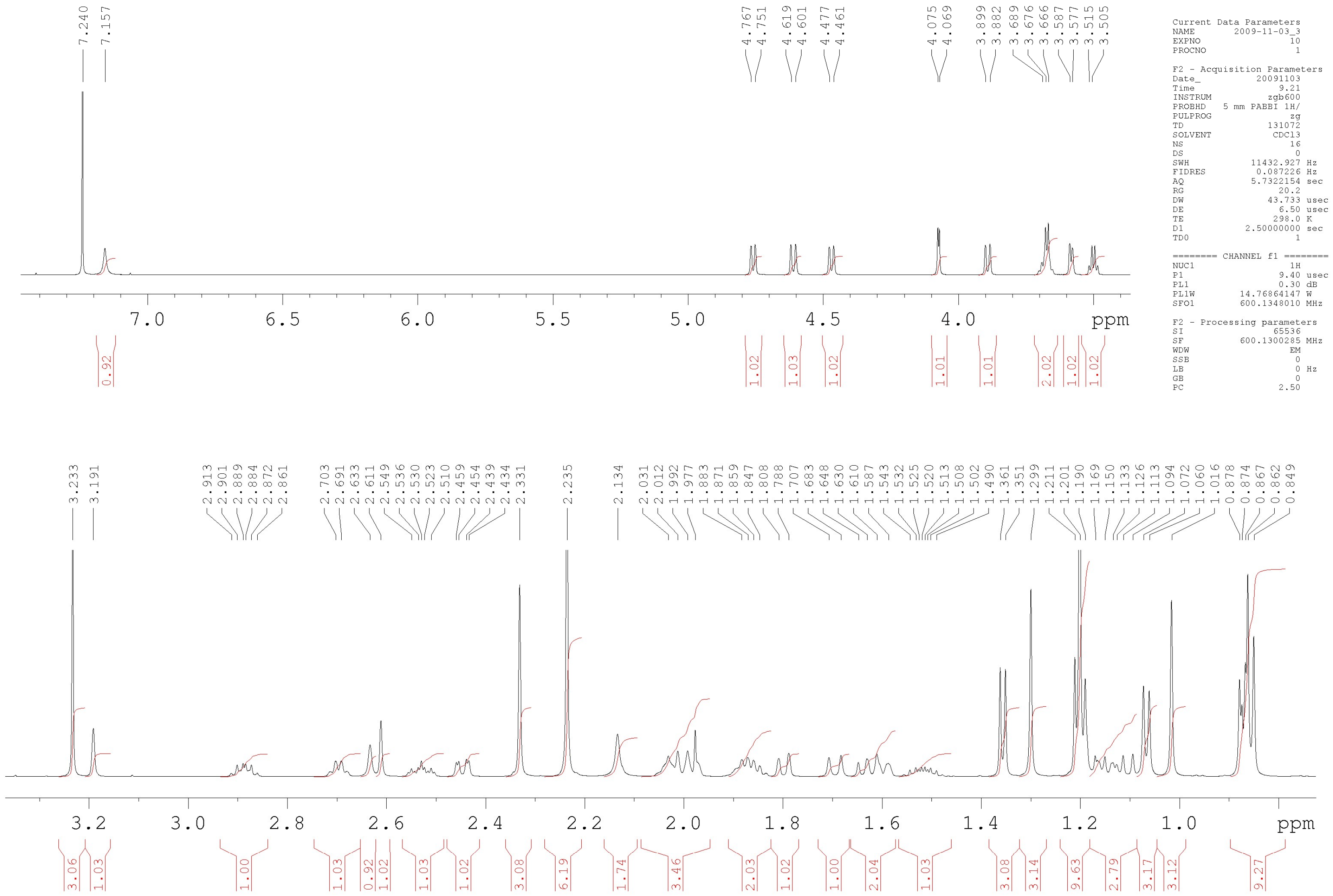

Figure S7. *^{1}H spectrum of **4** in $CDCl_3$ at 25 °C*

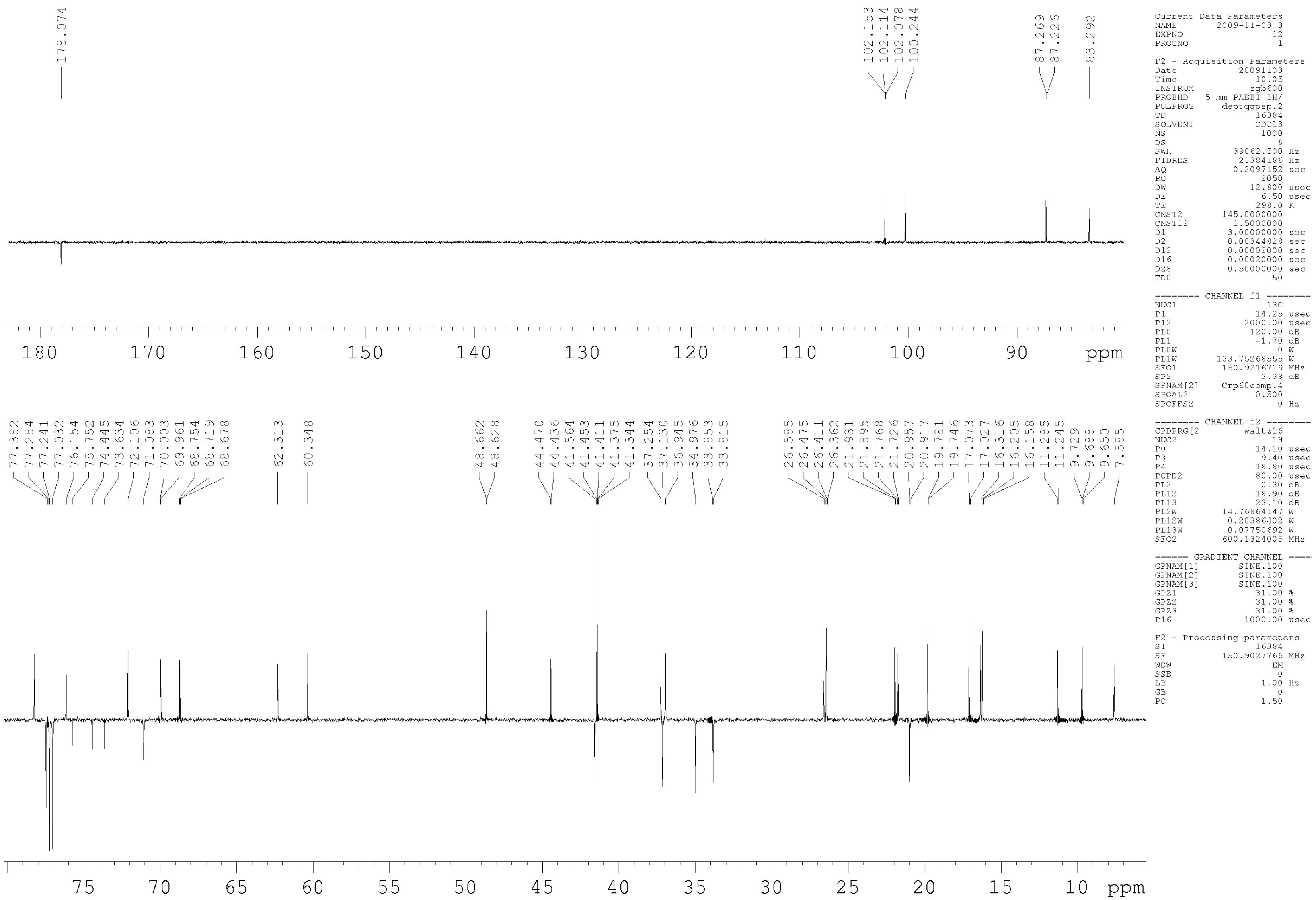

Figure S8. *^{13}C spectrum of **4** in $CDCl_3$ at 25 °C*

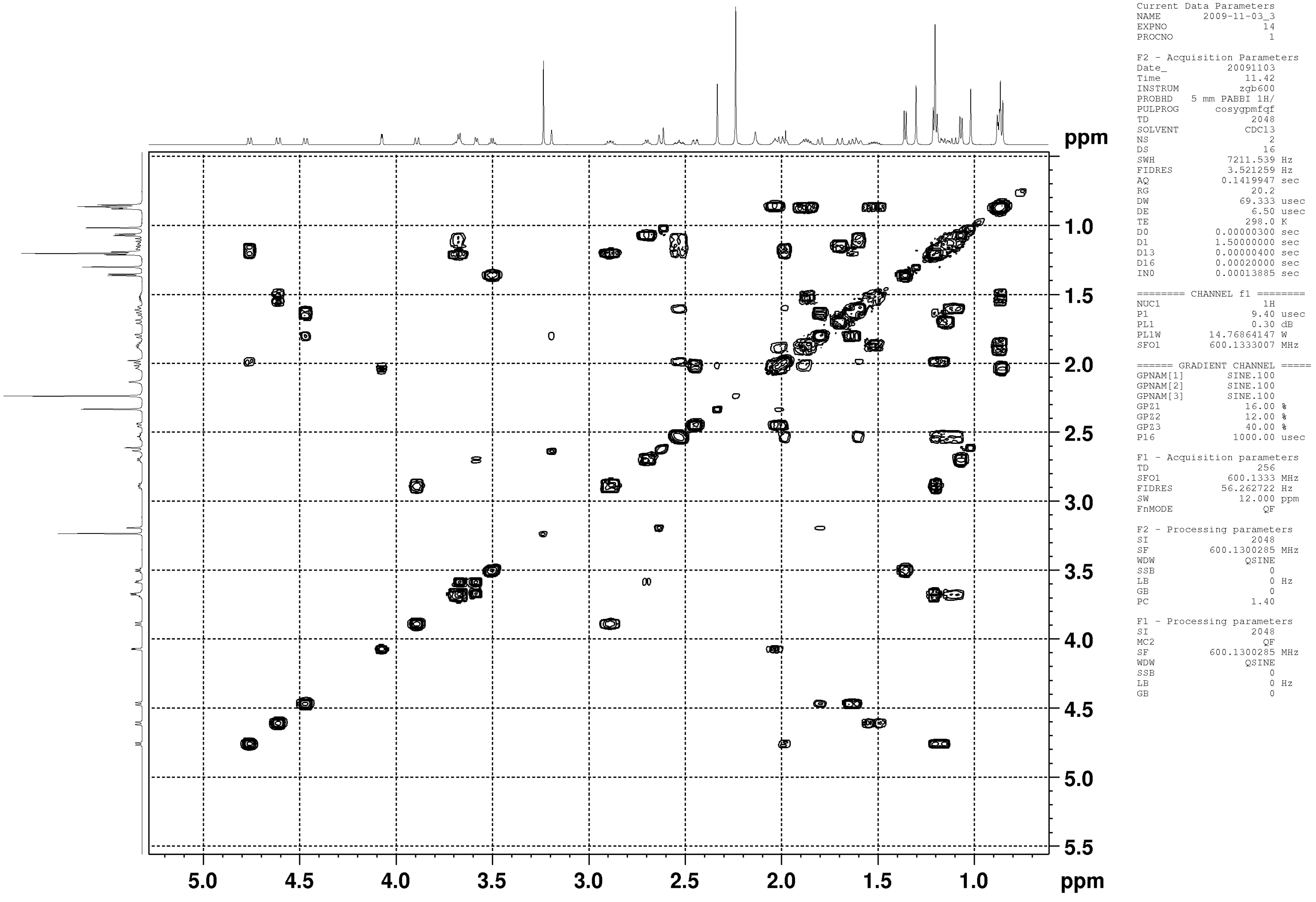

Figure S9. *^{1}H-^{1}H COSY spectrum of **4** in $CDCl_3$ at 25 °C*

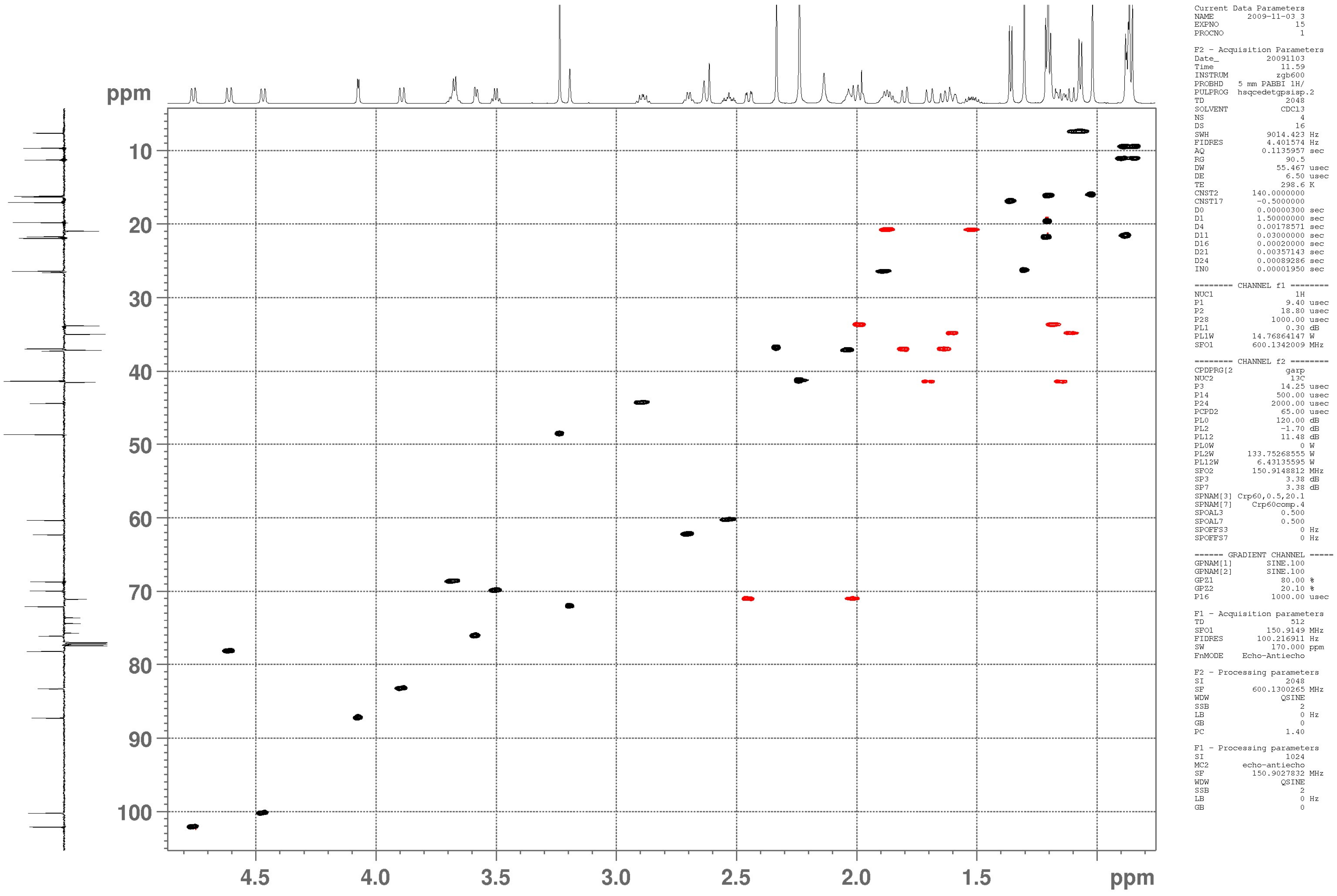

Figure S10. *^{1}H-^{13}C HSQCed spectrum of **4** in $CDCl_3$ at 25 °C*

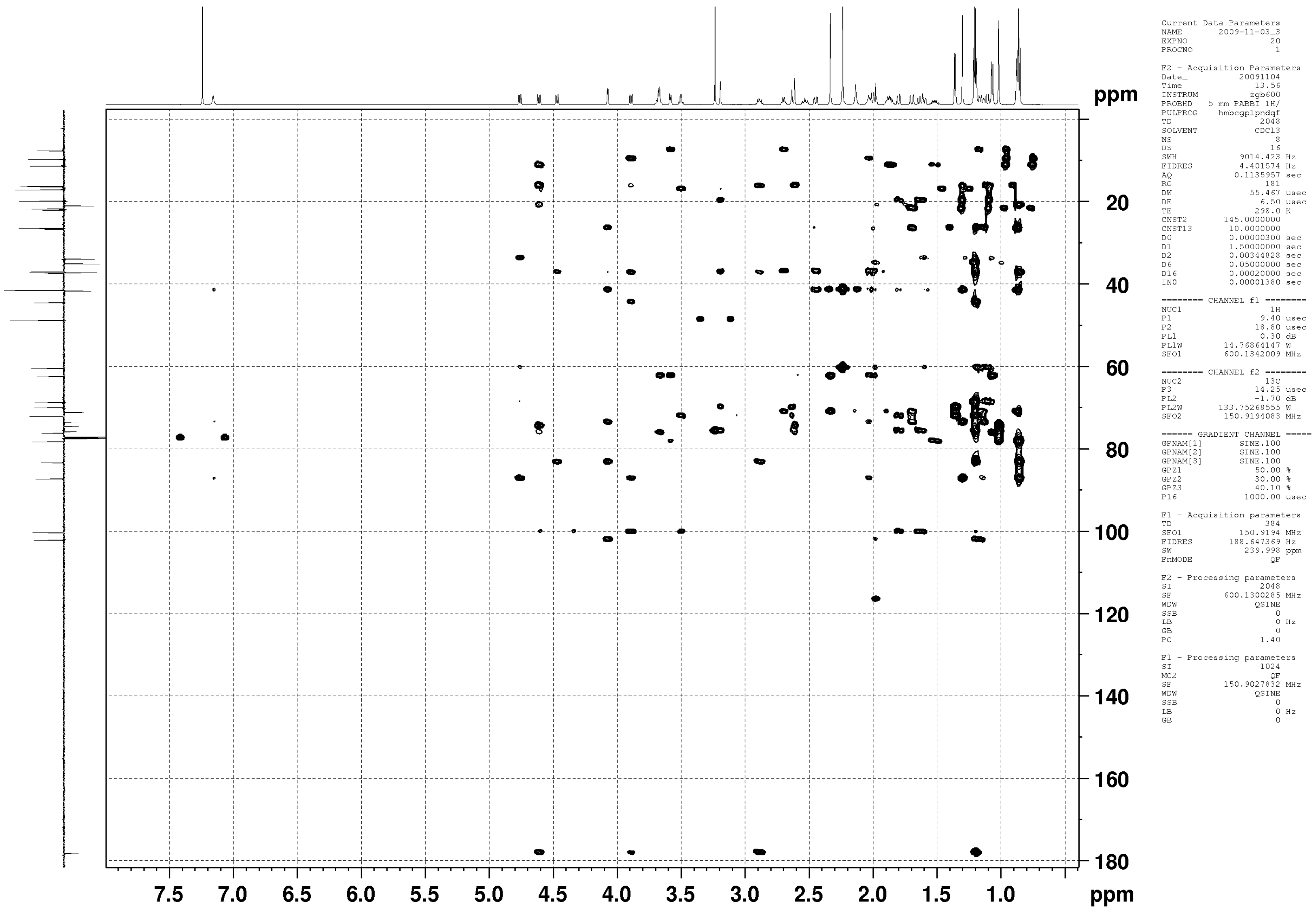

Figure S11. *^{1}H-^{13}C HMBC spectrum of **4** in $CDCl_3$ at 25 °C*

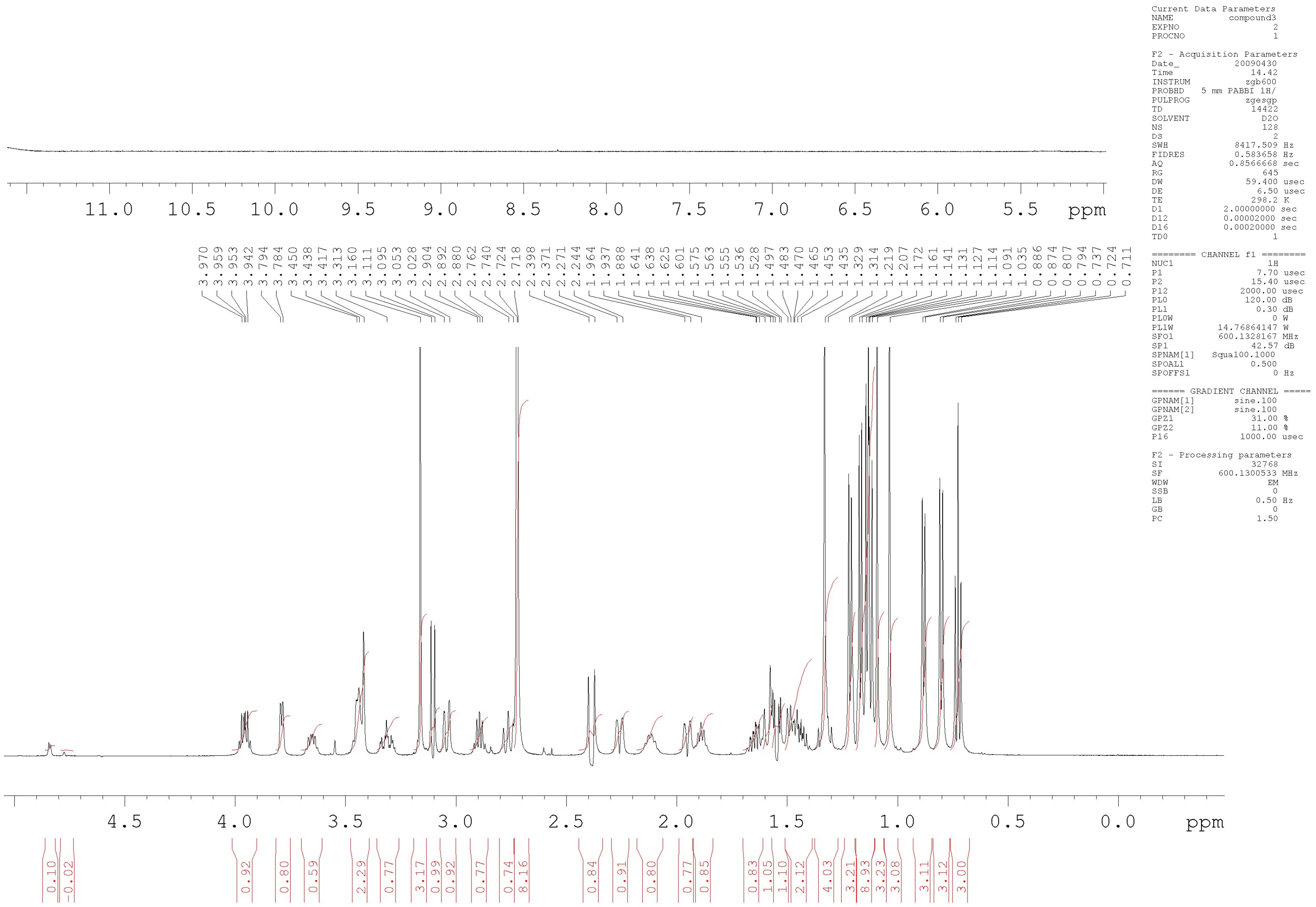

Figure S12. *Full* [1]*H spectrum of* ***3*** *in* D_2O *at 25 °C*

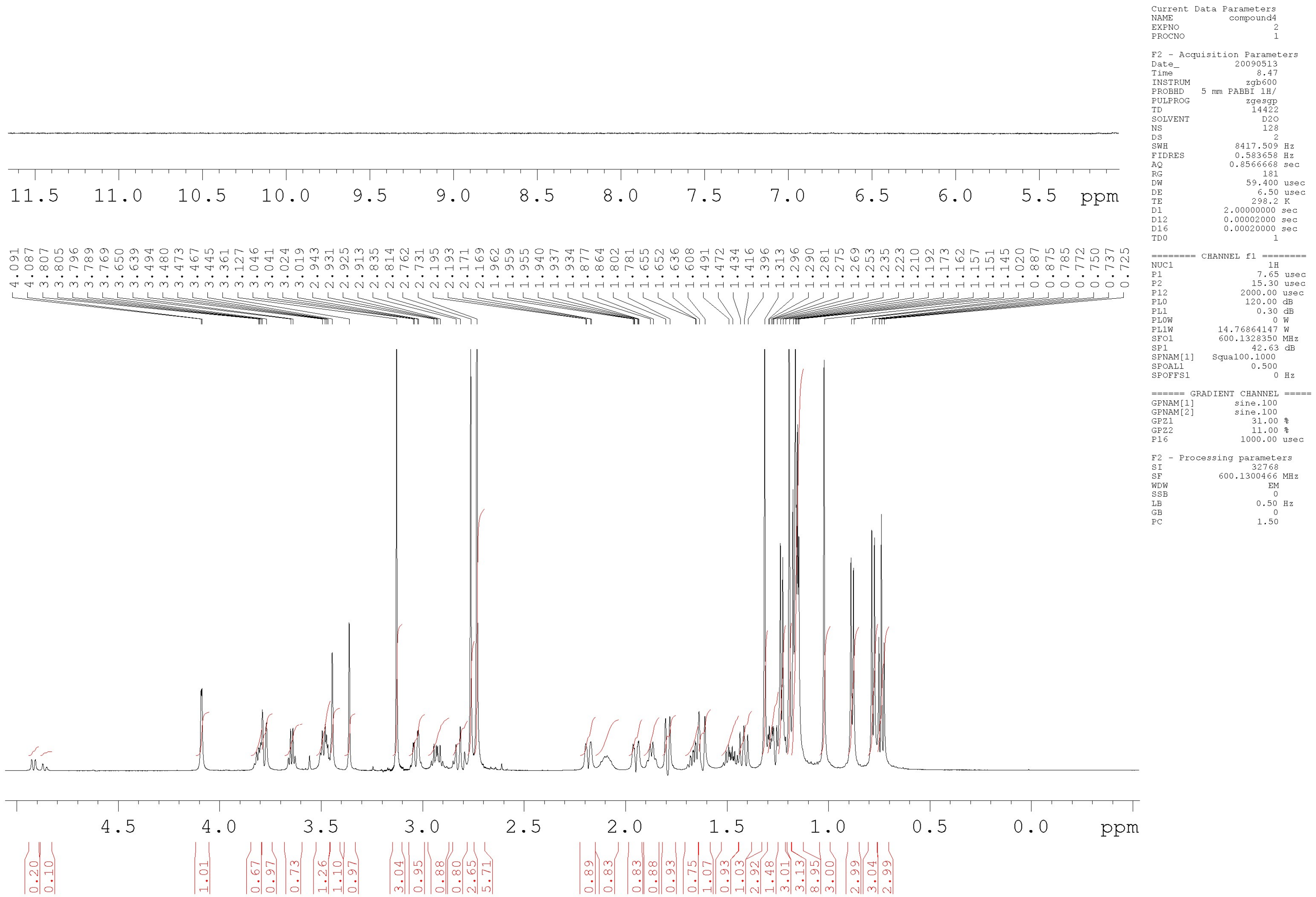

Figure S13. *Full ^{1}H spectrum of **4** in D_2O at 25 °C*

Table S1. *Comparison of nOe interactions (from ROESY) for **3** and **4** in D_2O at 25 °C; differences marked in red, overlap of 4'b, 5' CH_3 and 5'' CH_3 marked in yellow*

	2	2 CH_3	3	4	4 CH_3	5	6 CH_3	7a	7b	8	8 CH_3	9 NCH_3	9a	9b	10	10 CH_3	11	12 CH_3	13	14a	14b	15	1'	2'a	2'b	3'	3' NCH_3	4'a	4'b	5'	5' CH_3	1"	2"a	2"b	3" CH_3	3" OCH_3	4"	5"	5" CH_3
2	●			ss	s												m															ww							
2 CH_3		●	w		w																											w	s						
3		s	●	s	ww	s											s		ww				ww									m							
4	m		ss	●				m	s								s																						
4 CH_3	s				●	ww																	ww	m												s			
5			ss	m	ww	●	s		ww														ss															ss	
6 CH_3						ss	●	m		s	w																												
7a							m	●		w	s			w			ww														compound 3								
7b				m	ww				●		w			m			w																						
8							ss	w		●		ss	s	m																									
8 CH_3							ww	m		m	●	m	ss	m																									
9 NCH_3							w			s	ww	●	ss		s	s	s																						
9a										m	w	s	●		ww	m																							
9b									m				ss	●	s		s																						
10				w								s		ss	●			ss	w																				
10 CH_3												ss	w			●		m																					
11				ss					s					s			●	ss	ss																				
12 CH_3															s	w	ss	●	ww	ww	s																		
13																	ss		●	s	w																		
14a																			w	●																			
14b																		m			●																		
15																			ww			●																	
1'						ss																	●	ss		ss				ss						ss		ss	w
2'a					w																		s	●		s	s									w			
2'b																									●		s												
3'																							s	s		●	ss	ss		s					m				
3' NCH_3																								ss	s	ss	●	s											
4'a																										s	ss	●		s	m								
4'b						ww																	w				s		●										
5'																							s			ss		s		●								ss	
5' CH_3						ww																	w								●								
1"		s																												ss		●	s	ss			w	ww	
2"a																													ww		ww	ss	●		m	ss			
2"b																							ww			w	w					w		●	s		ss		
3" CH_3																																ss	m		●	ss	ss		
3" OCH_3		compound 4																															m	w	ss	●			
4"																													s		s			ww	ss	ss	●		s
5"																																ss			s		ss	●	
5" CH_3						ww																	w										ww				ss		●
